# Molecular Paleoparasitological Hybridization Approach as Effective Tool for Diagnosing Human Intestinal Parasites from Scarce Archaeological Remains

**DOI:** 10.1371/journal.pone.0105910

**Published:** 2014-08-27

**Authors:** Lauren Hubert Jaeger, Alena Mayo Iñiguez

**Affiliations:** Laboratório de Biologia de Tripanosomatídeos, Instituto Oswaldo Cruz, Fundação Oswaldo Cruz, Rio de Janeiro, Rio de Janeiro, Brazil; Institut Jacques Monod, France

## Abstract

Paleoparasitology is the science that uses parasitological techniques for diagnosing parasitic diseases in the past. Advances in molecular biology brought new insights into this field allowing the study of archaeological material. However, due to technical limitations a proper diagnosis and confirmation of the presence of parasites is not always possible, especially in scarce and degraded archaeological remains. In this study, we developed a Molecular Paleoparasitological Hybridization (MPH) approach using ancient DNA (aDNA) hybridization to confirm and complement paleoparasitological diagnosis. Eight molecular targets from four helminth parasites were included: *Ascaris* sp., *Trichuris trichiura*, *Enterobius vermicularis*, and *Strongyloides stercoralis*. The MPH analysis using 18^th^ century human remains from *Praça XV* cemetery (CPXV), Rio de Janeiro, Brazil, revealed for the first time the presence *E. vermicularis* aDNA (50%) in archaeological sites of Brazil. Besides, the results confirmed *T. trichiura* and *Ascaris* sp. infections. The prevalence of infection by *Ascaris* sp. and *E. vermicularis* increased considerably when MPH was applied. However, a lower aDNA detection of *T. trichiura* (40%) was observed when compared to the diagnosis by paleoparasitological analysis (70%). Therefore, based on these data, we suggest a combination of Paleoparasitological and MPH approaches to verify the real panorama of intestinal parasite infection in human archeological samples.

## Introduction

The first studies demonstrating the presence of parasites in human remains were of Ruffer [1], revealing the presence of *Schistosoma haematobium* in renal tissue of Egyptian mummies dating from 1250 to 1100 BC, and of Szidat [2], showing *Trichuris trichiura* and *Ascaris lumbricoides* eggs in well-preserved bodies from Prussia. The application of the rehydration technique of coprolites with trisodium phosphate [3], and the improvements of common parasitological diagnostic techniques [4] allowed considerable advances in paleoparasitology.

With the development of the PCR technique in the 1980s, the field of molecular paleoparasitology attracted interest from various research groups. Studies adjusting molecular techniques to the peculiarities of the archaeological material were published [5], [6]. *Ascaris* sp. [7], [8], *Trichuris trichiura* [9], and *Enterobius vermicularis* ancient DNA (aDNA) [10], [11] have been demonstrated in human remains by paleoparasitological analysis, as well as, by PCR and DNA sequencing. However, these techniques have some limitations that often do not allow a robust parasitological diagnosis. The low sensitivity of parasitological techniques, and the restrictions of applying PCR technique, due to DNA fragmentation/degradation and the presence of PCR inhibitors, complicates the diagnosis.

New approaches to improving the diagnosis of human parasites is essential to better understand the diseases plaguing in the past. Studies have shown the use of aDNA hybridization technique as an alternative tool to PCR for molecular paleoparasitological diagnosis [6], [12]. In this study, we present a new approach for molecular diagnosis of the main intestinal parasites in archaeological material in order to improve the diagnosis and to contribute to the knowledge of a real paleoepidemiology of these infections in the past.

## Material and Methods

### Ethics Statements

Sediment samples (XV24-XV33) from individuals buried on *Praça XV de Novembro* (November XV Square) used in this study belongs to the Paleogenetic collection of the *Laboratório de Biologia de Tripanosomatídeos* (LABTRIP/IOC/Fiocruz), under the responsibility of Dra. Alena Mayo Iñiguez, product of institutional collaboration with the Institute of Brazilian Archaeology (*Instituto de Arqueologia Brasileira*-IAB).

### The Archaeological Site *Praça XV* Cemetery (CPXV)

The city of Rio de Janeiro was founded in the early 16th century. It was an important commercial center for unrestrained Portuguese exploration. The *Praça XV de Novembro* was a central area of Rio de Janeiro city in colonial times. In this area, there was a port, where gold, diamond, coffee and sugar were commercialized in the Southeast region. The Rio de Janeiro port was also where Europeans and African slaves arrived in order to explorer the country and work the plantations, respectively. In 1996, during the construction of a tunnel in the port, human skeletons dating from the 18th and 19th centuries were discovered. It is known that there was a burial ground for African slaves who died in markets located near the port, as well as, the general population in the region. The archaeological campaign excavation was conducted by the IAB. During excavation, due to a high degree of disarticulation of the burials and the anatomic separation of individuals, complete skeletal series were not identified. Instead, a series of types of bones, skulls and mandibles for example, were collected, stored at room temperature, and protected from light. All bones were well preserved and were submitted to the curation process. In this process, bones were cleaned from the original soil, but no chemical treatment was performed.

### Precautions to Avoiding Contamination

Several precautions were taken to avoid aDNA degradation and DNA contamination during the collection procedure, and in aDNA analysis at Paleogenetic Laboratory (LABTRIP – IOC/Fiocruz). Clean protective clothing, gloves, head covering, mask and sterile material (tubes and instruments) were used. Paleoparasitologic experiments were performed under the procedures established for working with aDNA [13]. Sample preparation and aDNA extraction were performed at the Paleogenetic Laboratory. DNA hybridization, PCR positive controls, and electrophoresis were conducted at the main Laboratory (LABTRIP – IOC/Fiocruz). These two laboratories are physically distant from each other.

### Archaeological Samples

Sediment samples (n = 10) were recovered from the interior of the sacral foramina of ten individuals by scraping, as described [14]. Sediment scrapings from the interior of skulls were used as negative controls for paleoparasitological analysis. Samples were transported to laboratory at 4°C and kept at −20°C until paleoparasitological and paleogenetic analysis.

### Ancient DNA Extraction

The sediment samples were rehydrated (1∶2 w/v) with 0.5% aqueous trisodium phosphate solution for 48 hours at 4°C. About 200 µL sediment solution was used for aDNA extraction. Samples were treated with 1.2 mL digestion buffer (NaCl 10 mM, Tris-HCl 10 mM, SDS 0.5%, EDTA 50 mM, pH 8.0) with 1 mg/mL of Proteinase K (Invitrogen) and incubated at 56°C for 24 hours. Thereafter, samples were treated with IQ System (Promega) according to manufacturer's instructions. The aDNA was purified by a GFX PCR DNA and Gel Band Purification kit (GE HealthCare) and the DNA concentrations were estimated on spectrophotometer.

### DNA Probes of Parasites

Modern fecal samples were used to set up the positive controls for preparing the hybridization probes. Eight molecular targets from four helminth parasites were included: *Ascaris* sp., *T. trichiura*, *E. vermicularis*, and *Strongyloides stercoralis*. Human and skull sediment DNA were included as negative controls. The DNA extraction were performed with phenol:chloroform method and purified by a GFX PCR DNA and Gel Band Purification kit (GE HealthCare). PCRs were performed as described for each target (Table 1). PCR primers were designed to target specific fragments of the 18S rDNA gene from *T. trichiura* (DQ118536), and the *cox*I gene from *E. vermicularis* (AB221474), using program PRIMER3 (http://www.bioinformatics.nl/cgi-bin/primer3plus/primer3plus.cgi/). PCR conditions for primers designed in this study were: PCR final volume of 50 µl using 5X PCR buffer (20 mM Tris-HCl, 0.5 µM KCl), 2.5 mM MgCl_2_, 0.4 mM each dNTPs, 100 ng of each primer, 1.0 U of Platinum *Taq* (Invitrogen) and 50–100 ng DNA. The reactions were subjected to an initial cycle of 5 min at 96°C, followed by 35 cycles of 96°C for 1 min, 50–55°C for 30 sec and 72°C for 60 sec in a programmable thermal controller (Eppendorf Mastercycler personal). Extraction and negative PCR controls were included. PCR products were visualized by 2–3% agarose gel electrophoresis stained with ethidium bromide. PCR products were directly sequenced using Big Dye Terminator v 3.1 Cycle Sequencing Ready Reaction kit (Applied Biosystems) in a 3100 Automated DNA Sequencer as recommended by the suppliers. Pairwise/Blast/NCBI command, Chromas v 1.45 and BioEdit v7.0.1 software were used for editing and sequence analysis. Sequencing and sequence analysis were performed to confirm the molecular target of each parasite, before conducted the aDNA hybridization. All the experiments with modern DNA and positive controls were carried out at the main Laboratory (LABTRIP – IOC/Fiocruz), physically distant from the Paleogenetic Laboratory.

**Table 1:** Molecular targets used in the Molecular Paleoparasitological Hybridization (MPH).

Parasite	Molecular target	Primer sequence (5′-3′)	pPCR size	Reference
*Ascaris* sp.	*cyt* b	CCRB Asc1 GTTAGGTTACCGTCTAGTAAGG	142	[7]
		CCRB Asc2 CACTCAAAAAGGCCAAAGCACCC		
	*nad* I	MH5F TATGAGCGTCATTTATTGGG	∼400	[25]
		As-NDR GCATCACAATAGCCAACAAATAC		
*Trichuris trichiura*	18S rDNA	TT0926F TTGCGAAAGCATTTGTCAAG	680	Designed in this study
		TT1606R ACGTTTCAACCGATTTCCTG		
	18S rDNA	TT1315F CGAACGAGACTCTGGCCTAC	∼390	Designed in this study
		TT1709R GTACAAAGGGCAGGGACGTA		
*Enterobius vermicularis*	*cox* I	EvPRA GGAGGTGTTTGGTCATTTGG	198	Designed in this study
		EvPRB CGTCCCCCTATCAAAGTCAA		
	5S rDNA SR	Entf CACTTGCTATACCAACAACAC	420	[6]
		Entr GCGCTACTAAACCATAGAG		
*Strogyloides stercoralis*	18S rDNA	Stro18S-1530F GAATTCCAAGTAAACGTAAGTCATTAGC	101	[26]
		Stro18S-1630R TGCCTCTGGATATTGCTCAGTTC		
	28S rDNA	StroS TTAGAGTCGTGTTGCTTGGAA	180	[27]
		StroAS GTGCAACTGGCTCTGTATGC		

Abbreviations: bp, base pair; pPCR, PCR product; *cyt* b, cytochrome b; *nad* I, NAD dehydrogenase; ITS, Internal transcribed spacer; cox I, cytochrome c oxidase subunit I; rDNA, ribosomal DNA; SR, Spacer region.

### Ancient DNA Hybridization Analysis

A dot blot procedure was conducted as described elsewhere [15]. DNA probes were prepared by PCR, purified by GFX PCR DNA and Gel Band Purification kit (GE HealthCare) and labeled by chemiluminescence using Gene Images Alkphos Direct Labeling and Detection Systems (Amersham), according to manufacturer's instructions.

## Results

Ten samples of individuals from the archaeological site CPXV were analyzed by MPH. Three helminthes were detected in in these samples: *T. trichiura*, *Ascaris* sp., and *E. vermicularis* (Table 2). We observed aDNA of *T. trichiura* and *Ascaris* sp. in 40% of the samples and *E. vermicularis* in 50%, while *S. stercoralis* aDNA was not detected. Positive controls had results of strong intensity of chemiluminescence. Chemiluminescence signals were not detected in negative controls.

**Table 2:** Results of the Molecular Paleoparasitological Hybridization from archeological site *Praça XV* cemetery, Rio de Janeiro, Brazil.

Sample	Paleoparasitological Analysis a				Molecular Paleoparasitological Analysis			Prevalence of Infection	
	*Ascaris* sp.	*Trichuris trichiura*	*Enterobius vermicularis*	*Taenia* sp.	*Ascaris* sp.	*Trichuris trichiura*	*Enterobius vermicularis*	*Ascaris* sp.	*Trichuris trichiura*
XV24	−	+	−	−	+	−	−	+	+
XV25	−	+	−	−	+	+	+	+	+
XV26	−	+	−	−	−	−	−	−	+
XV27	−	−	−	−	−	−	−	−	−
XV28	−	+	−	−	−	−	−	−	+
XV29	−	+	−	−	−	+	+	−	+
XV30	−	−	−	−	−	−	−	−	−
XV31	+	+	−	−	−	−	+	+	+
XV32	−	+	−	−	+	+	+	+	+
XV33	−	−	−	+	+	+	+	+	+
n = 10	1 (10%)	7 (70%)	0	1 (10%)	4 (40%)	4 (40%)	5 (50%)	5 (50%)	8 (80%)

*Strongyloides stercoralis* negative results from this study were omitted.

a results from [14].

Abbreviations: +, positive result; −, negative result.

## Discussion

Deguilloux and colleagues [16] state: “Cemeteries potentially hold a wealth of information about the biological and social aspects of the communities who used them.” The archaeological site CPXV was a mass grave that was used to bury African slaves and inhabitants in general who died as a result of epidemics that plagued the city during the colonial period. It is known that Rio de Janeiro city was the gateway of the country during the colonial period. It was the capital of Brazil (1763 to 1960), and became the main commercial center of the country, that as a consequence underwent massive urbanization [17]. However, urban and population growth was accompanied by adequate public hygiene policy.

A former study in CPXV archaeological site demonstrated the presence of *T. trichiura, Ascaris* sp. and *Taenia* sp. eggs by light microscopy [14]. In the present study, we detected aDNA of *T. trichuira* and *Ascaris* sp. using the MPH approach. These helminthes are transmitted through soil contaminated by human feces, via the fecal-oral route, and reflect poor sanitary conditions of populations [18]. *T. trichuira* and *Ascaris* sp. infections occurs concomitantly and are the most frequent intestinal parasites found in current and archaeological fecal samples. These helminthes were described in Brazilian archaeological sites dating from pre-Columbian [19], [20], [8] and historical periods [14], [21]. The present study confirms the presence of *T. trichiura* and *Ascaris* sp. infections in individuals from the CPXV archaeological site. Although sediment samples were collected from skeletons that had been submitted to museological curation, it was possible to recover parasitic forms and aDNA. This study confirms the importance of using specimens from museum collection in paleogenetic analysis, even after going through a curation process.

Our results showed for the first time the presence of *E. vermicularis* aDNA in an archaeological site from Brazil. *E. vermicularis* aDNA was detected in 50% of individuals However no pinworm eggs were visualized by parasitological techniques. These results are not surprising, since previous studies have shown the presence of aDNA of pinworm in coprolites without eggs visualization [10], [11]. This fact suggests that environmental conditions of the city and taphonomic factors affect more helminth eggs, such as *E. vermicularis*, than others, such as *Ascaris* sp. and *Trichuris* sp.. *Ascaris* sp. and *Trichuris* sp. eggs have multiple layers and resistant protective proteins allowing a greater resistance to external environment, opposite to the slight eggs of pinworms. The lightweight and sticky eggs characteristic of pinworm, are also an advantage for the maintenance of direct transmission between human hosts. Considering the life cycle of pinworm, pregnant females release the eggs in the perianal region, which could be carried by the feces. However, these eggs are not usually observed in conventional parasitological examinations. Even using enrichment parasitological techniques, fecal examination reveals only 5–10% of enterobiasis cases [22]. Molecular paleoparasitological analysis can overcome this issue, allowing identification or confirmation of an infection even in the absence of visible parasite forms [6], [8], [11], [23].

*Strongyloides stercoralis* infection was not detected in this study by MPH approach. Through paleoparasitological techniques *S. stercoralis* was observed in archaeological sites from Egypt, USA and The Netherlands [18]. But, it was not diagnosed by paleogenetic techniques. Considering the life cycle of this parasite, eggs are normally released inside the host intestine. The larvae in feces require a period in the soil to become infective [22]. Larvae are markedly more fragile structures than eggs, and does not resist very well to the effects of time and/or the taphonomic process.

A Molecular Paleoparasitological approach was developed using aDNA hybridization technique with two specific targets for each parasite. Previous studies have demonstrated the detection of pathogens in archaeological remains using this technique [6], [12]. This seems to be the ideal technique in paleogenetic analysis, especially working with poorly preserved samples. Moreover, this assay has advantages over PCR: (i) it is highly sensitive to detect fragments of aDNA; (ii) it is not affected by the action of inhibitors of the enzymatic reaction, that are common in aDNA from archeological samples; (iii) it is not affected by molecules and cross-linking processes, that block *Taq* DNA polymerase in PCR amplification; (iv) the aDNA membrane could be reused with different targets, allowing the optimization of use of the original samples [15].

We detected aDNA in samples that were negative in previous paleoparasitological analysis [14]. Parasitic forms of helminth eggs and protozoan cysts could not be visualized by light microscopy, possibly because they did not resist the action of taphonomic process. Another possibility is that the infection could also be present a low parasite load. However, CPXV individuals have demonstrated 80% of the prevalence of parasites infection [14]. In the present study, *Ascaris* sp. and *E. vermicularis* prevalence increased considerably when aDNA hybridization was applied. Interestingly, one individual that was positive only for *Taenia* sp., it was also positive for *Ascaris* sp., *T. trichiura* and *E. vermicularis* aDNA after the MPH application. These results are coherent with the source of material examined, which was cleaned from human remains. As mentioned, in the first analysis conducted in the CPXV site, the paleoparasitological results represented an underestimation of the prevalence of intestinal infection. We also had stated that higher intensity, and/or diversity of recovered parasites would likely be expected, if it was possible to access all organic material. The MPH approach applied here was a better way to optimize the investigation of original samples in order to access the real status of infection.

On the other hand, negative results for aDNA were observed in samples with positive paleoparasitological results. The *post-mortem* instability of nucleic acids is the methodological problem inherent in aDNA research [24]. Moreover, is common to find helminth eggs without content inside, as observed in individuals buried in CPXV [14]. We found a large number of *T. trichiura* eggs (n = 26), but most of them possessed no content or polar plugs. This explains the low aDNA detection of *T. trichiura* (40%) in contrast to paleoparasitological analysis (70%). Most importantly, these results demonstrated once again the relevance of traditional paleoparasitology in diagnosis by the observation of parasites and/or parasitic structures in archeological samples, when aDNA is not preserved.

The results presented here revealed that the application of paleoparasitological and MPH approaches together allowed us to access the real panorama of intestinal infection in archeological samples. When performing these assays independently, differences in the infection prevalence were observed. Therefore, we recommend the use of MPH approach with the aim of confirming and complementing the diagnosis of human intestinal parasites in archeological samples.
